# Test, Trace, and Put on the Blockchain?: A Viewpoint Evaluating the Use of Decentralized Systems for Algorithmic Contact Tracing to Combat a Global Pandemic

**DOI:** 10.2196/26460

**Published:** 2021-04-06

**Authors:** Moritz Platt, Anton Hasselgren, Juan Manuel Román-Belmonte, Marcela Tuler de Oliveira, Hortensia De la Corte-Rodríguez, Sílvia Delgado Olabarriaga, E Carlos Rodríguez-Merchán, Tim Ken Mackey

**Affiliations:** 1 Department of Informatics King's College London London United Kingdom; 2 Department of Neuromedicine and Movement Science Faculty of Medicine and Health Sciences Norwegian University of Science and Technology Trondheim Norway; 3 Department of Physical Medicine and Rehabilitation Hospital Central de la Cruz Roja San José y Santa Adela Madrid Spain; 4 Department of Epidemiology and Data Science Amsterdam UMC Amsterdam Netherlands; 5 Department of Physical Medicine and Rehabilitation La Paz University Hospital Madrid Spain; 6 Department of Orthopaedic Surgery La Paz University Hospital Madrid Spain; 7 Osteoarticular Surgery Research Hospital La Paz Institute for Health Research, IdiPAZ Madrid Spain; 8 Department of Anesthesiology, Division of Infectious Diseases and Global Public Health School of Medicine UC San Diego La Jolla, CA United States; 9 BlockLAB San Diego Supercomputer Center UC San Diego La Jolla, CA United States

**Keywords:** COVID-19, public health, blockchain, distributed ledger technology, mobile apps, pandemic mitigation, contact tracing, epidemiological monitoring

## Abstract

The enormous pressure of the increasing case numbers experienced during the COVID-19 pandemic has given rise to a variety of novel digital systems designed to provide solutions to unprecedented challenges in public health. The field of algorithmic contact tracing, in particular, an area of research that had previously received limited attention, has moved into the spotlight as a crucial factor in containing the pandemic. The use of digital tools to enable more robust and expedited contact tracing and notification, while maintaining privacy and trust in the data generated, is viewed as key to identifying chains of transmission and close contacts, and, consequently, to enabling effective case investigations. Scaling these tools has never been more critical, as global case numbers have exceeded 100 million, as many asymptomatic patients remain undetected, and as COVID-19 variants begin to emerge around the world. In this context, there is increasing attention on blockchain technology as a part of systems for enhanced digital algorithmic contact tracing and reporting. By analyzing the literature that has emerged from this trend, the common characteristics of the designs proposed become apparent. An archetypal system architecture can be derived, taking these characteristics into consideration. However, assessing the utility of this architecture using a recognized evaluation framework shows that the added benefits and features of blockchain technology do not provide significant advantages over conventional centralized systems for algorithmic contact tracing and reporting. From our study, it, therefore, seems that blockchain technology may provide a more significant benefit in other areas of public health beyond contact tracing.

## Introduction

### Background

To many global health professionals, the emergence of the COVID-19 pandemic has not come as a complete surprise. The outbreak of SARS that occurred in the autumn of 2002 in Guangdong Province, China—characterized as the first near-pandemic in the era of globalization [1]—marked the beginning of a new century in which global health security events would become more frequent and escalate rapidly across the globe. Following SARS came the global pandemic of H1N1, outbreaks of Middle East Respiratory Syndrome, the Zika virus, and new Ebola outbreaks. These served as early warning signs of what would become the most significant human health emergency since the 1918 influenza pandemic: the current COVID-19 global pandemic that has, as of February 2021, resulted in at least 100 million cases and 2 million deaths worldwide [2].

Throughout this period of accelerated outbreaks of novel, emerging, and re-emerging infectious diseases, calls for sound public health policy and further expansion of public health surveillance capacity to prevent future pandemics have become more frequent and urgent [3]. However, investment in public health infrastructure, such as strengthening state capabilities under the World Health Organization International Health Regulations, did not heed these warnings: experts have painted a bleak picture of outbreak preparedness by characterizing the global pandemic response as cycles of panic succeeded by neglect [4]. Consequently, various systems for disease surveillance, including electronic public health reporting modalities, were challenged by the complex requirements associated with COVID-19-related data. Some of these challenges stemmed from the inability of the relevant public health agencies to receive and share electronic data at a pandemic scale [5], some from the use of inappropriate or outdated tools lacking interoperability [6], and some from failing to meet security and privacy requirements [7].

As COVID-19 cases continue to surge, national governments have attempted to invest in and deploy more robust digital disease surveillance systems. These encompass different forms of technology (eg, digital epidemiology, big data, machine learning, mobile apps, and distributed computing), which are now viewed as critical tools to explore in order to modernize the pandemic response [8]. While a rapid increase in innovation and investment in this area of technology has occurred, many of these technology-centric initiatives have encountered implementation barriers due to nontechnical challenges associated with data governance, user adoption, concerns about accountability and oversight, and patient privacy and social acceptance concerns [7,8]. An emerging technology that has been suggested in this context is blockchain, a form of distributed ledger technology that is maturing in several industries, including in areas of digital cryptocurrencies, financial transaction technology, and growing attention in industrial sectors, such as energy, transportation, supply chain, auditing, and health care [9].

### Blockchain Uses in Health Care

The adoption of blockchain, which can be characterized as an append-only distributed database that is coordinated via a peer-to-peer protocol [10], removes the need for central operators and can offer potential improvements over traditional health care information management systems (eg, client-server systems) [9]. Blockchain allows for tamper-proof replication of data in an adversarial environment [11]. The technology is resilient to fault scenarios in which adversaries send conflicting information to different parts of the system [12], even if those adversaries present large numbers of pseudonymous identities with malicious intent [13]. Participants on a blockchain form consensus on whether a proposed record is admissible by adjudicating it using a consensus mechanism [14], thus ensuring only valid records agreed upon by network members are replicated.

Consensus on a blockchain network can be proof based (eg, proof-of-work consensus as used by the Bitcoin blockchain) or voting based (eg, proof-of-authority consensus) [15], with different hybrid forms being an emerging field of research [16]. Regarding access control, blockchain protocol taxonomies differentiate between public or private and permissionless or permissioned networks [17]. Public blockchains are open to participation by anyone, whereas private, or *enterprise*, blockchains employ access control mechanisms. In a permissionless system, all members have the same responsibilities in the consensus protocol, while permissioned networks assign different responsibilities in consensus to participants, depending on their role and authority.

Several use cases have emerged evidencing the potential utility of blockchain in health care data management. These include electronic health record (EHR) management and aggregation, privacy-preserving algorithms for health systems data, integration of blockchain systems with the Internet of Medical Things, enabling distributed patient-provider directories across multiple payers and providers, and enhancing management and security of health supply chains [18-21]. Accompanying this potential, blockchain also faces real-world implementation challenges, including storing and transferring data on- and off-chain, interoperability with other health information systems, managing permission structures, and ensuring scalability [22].

Blockchain has also been suggested as a potential solution in the context of COVID-19 algorithmic contact tracing by promising protection from cyberattacks [23], allowing for global monitoring of social encounters to inform health policies [24], enabling privacy [25,26], preventing the falsification of diagnoses [27], allowing users to retain ownership of personal data [28], and ensuring the trustworthiness of that data [29], while maintaining a record of its provenance [26]. While none of the popular algorithmic contact tracing frameworks on the market today [30] uses blockchain, the growing number of academic works [23,25,27,31-38] suggests significant interest. Hence, this viewpoint aims to critically examine the potential utility and technical feasibility of blockchain technology for pandemic algorithmic contact tracing. This is accomplished by applying a blockchain evaluation framework that assesses the suitability of using the technology for specific use cases based on seven key questions. The viewpoint concludes with some recommendations of whether blockchain is a viable application for this critical public health use case and other observations about how to leverage this technology in the ongoing fight against COVID-19.

## Algorithmic Contact Tracing

### Overview of Conventional and Algorithmic Contact Tracing Approaches

Contact tracing is an epidemiological control measure aimed at identifying all the people with whom an individual who contracted an infectious disease has been in contact, and who are, in turn, at risk of being infected with and transmitting that disease to other close contacts [39]. It has pronounced benefits in controlling infections that remain undetected in the population [40], such as the transmission of COVID-19, in which a large proportion of cases could be asymptomatic [41]. Quick, reliable, and accurate tests to confirm cases are a prerequisite for successful contact tracing [42,43], as, without them, infectious individuals can remain unidentified and continue to serve as human vectors sustaining community transmission. Insufficient testing can lead to underreporting of the true prevalence of the disease and its attack rate, as well as limit the effectiveness of nonpharmaceutical interventions, such as masking, social distancing, and other crucial public health interventions [44]. Contact tracing, however, represents only one single stage in the process of effective outbreak control and response, which is only effective when combined with quarantine and isolation procedures [45].

Contact tracing has a rich history dating back to the late nineteenth century, when UK medical officers responded to infectious disease outbreaks such as smallpox with surveillance systems involving notification, isolation, disinfection, and case finding [46]. The information age brought digital case management systems and other innovations (eg, digital epidemiology via mobile apps, internet surveillance, and disease modeling and forecasting using artificial intelligence) that are now being leveraged by health authorities. Yet, traditional interview-based approaches remain a mainstay [8,47,48]. Here, contact information is collected by health care professionals or volunteers who discover the contact history of individuals affected by an infectious disease through interviews with patients, families, or health care professionals or by analyzing medical records, tracking data, or surveillance data [49].

Where an interview-based contact tracing technique is used, its success relies on the ability of those affected to recollect their contact history. The reliability of such self-reported data is, however, questionable [50]. Moreover, contact with unknown persons cannot be discovered through this approach. Furthermore, conventional contact tracing regimens are labor intensive, are associated with high costs per case, and yield diminishing disease prevalence reductions under incremental investments [51]. Consequently, doubts have been raised about whether these traditional methods alone can be effective in the context of a large-scale pandemic. As a reaction, digital epidemiology methods, including algorithmic contact tracing, have been proposed to reduce virus transmission more effectively [8]. Digital epidemiology, or “the use of data generated outside the public health system for disease surveillance” [8], has been discussed since the 1990s [52]. Most of the early approaches to digital epidemiology use *passive* methods by repurposing data from “a range of sources most of which do not relate to healthcare utilization” [supplementary material, 8]. In contrast, most modalities of algorithmic contact tracing can be considered *active* methods, as users have to enable data monitoring and sharing consciously.

Algorithmic contact tracing automates the conventional contact tracing process by allowing for the collection, aggregation, and analysis of automatically generated data about a case’s contacts by a public health agency, thus eliminating the need for laborious interviews. The large volume and multimodal nature of the clinical informatics systems and epidemiological data that go along with this approach constitute a challenge that new technologies can help address [53]. This applies particularly to algorithms that can quickly assess potential exposure and risk patterns while enabling faster notification to suspected contacts [54]. Countries that have applied algorithmic contact tracing aggressively, by making the use of mobile phone tracing apps compulsory (eg, China and South Korea), were able to reduce daily positive cases more effectively than those that used approaches where participation was voluntary [55]. While it is unclear whether the containment of case numbers can be directly attributed to algorithmic contact tracing, the use of big data to trace individuals is a commonality of the pandemic containment strategies applied by these countries [56]. Further, real-world experiences with the deployment of algorithmic contact tracing illustrate the complexity of the ethical issues associated with these technologies, including the need to balance individual privacy and autonomy concerns with the utility of such data to prevent disease spread during a public health emergency [8,56].

To reiterate, the goal of contact tracing is to identify people that *had contact* with each other, thereby identifying a potential path of exposure and infection. While a definition of what can be considered *contact* in the context of COVID-19 is still evolving [57], active algorithmic contact tracing commonly uses physical proximity and duration of exposure [58] as an approximation. Data gathered for algorithmic contact tracing commonly takes one of three forms: (1) proactively reported data (ie, manual digital check-ins that require participants’ compliance [59]), (2) active sensor data (ie, information about an encounter with a different device utilizing the same tracing app), and (3) passive sensor data (ie, information about the geographic position of the device). These are commonly generated by devices using Bluetooth, including Bluetooth Low Energy; GPS; and Wi-Fi signal strength information [60]. Bluetooth allows for active sensing, delivering information about the proximity of two sensors with submeter accuracy [61,62]. Passive sensing through GPS and Wi-Fi uses environmental data to approximate the geographic position of a device with relatively high precision under good conditions [63,64], but insufficient precision under suboptimal conditions [65-67]. Based on this information, an algorithmic contact tracing system can reconstruct when individual devices have been close, thereby allowing it to proactively alert those who are identified as being at risk of infection once confirmation of a positive case is made known to the system.

### Existing Literature on Blockchain Technology for Algorithmic Contact Tracing

As previously discussed, algorithmic contact tracing is now an emerging use case for blockchain technology. However, little had been published on this topic and its application to infectious disease control before the COVID-19 pandemic, despite prior outbreaks of other diseases. One of the first contributions in this space is by Kangbai et al [68], who proposed a “Blockchain platform to conduct real-time Ebola contact tracing” in the context of the 2018 outbreak of this highly virulent virus in the Democratic Republic of Congo. Subsequently, the COVID-19 pandemic has led to increased interest in the topic and several publications and preprints in the medical and engineering literature. From a systemic perspective, the dominant function of blockchains in algorithmic contact tracing is that of tamper-proof, distributed data stores for managing contact data [23,25,27,31-38]. A less pronounced function is that of a data integration layer that allows for the exchange of medical and public health information from different sources among different actors in health care settings [23,25,27,32]. Data that are exchanged in this way take the shape of certified COVID-19 diagnostic data [23,25,32] or immunization data [27].

**Figure 1:**
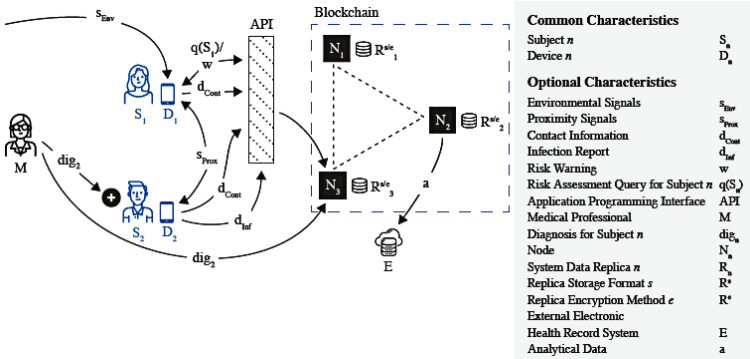
An archetype of a blockchain-based contact tracing environment derived from architectures described in the published literature. EHR: electronic health record.

Proposed algorithmic contact tracing blockchain systems appear as distributed architectures consisting of varying numbers of nodes in a network (see Figure 1). Commonly, these systems do not employ access control but, instead, grant read and write access to the public [23,25,31,33-36,38]. Less commonly, read access is provided to the public but write access is restricted [37], yet other architectures operate as private systems [27,32]. In most public systems, each node stores a replica of all data network-wide. Here, data are potentially encrypted and stored according to a format specific to the protocol used. In some architectures, external entities like hospitals or laboratories access the blockchain to obtain data for analysis [37], potentially correlating them with data held by external EHR systems. The consensus mechanisms used for data replication between nodes are rarely discussed. Where they are, mechanisms are selected either for their performance characteristics [25] or to implement authority-based forms of consensus [23,35,37]. While some proposals do not discuss the role of smart contracts [31,37,38], many employ smart contracts to validate data on the chain [23,32,34-36], mostly to prevent malicious users from inserting fabricated records of positive diagnoses into the system [23,32].

Proposed blockchain-based algorithmic contact tracing systems (see Figure 1) also generally cater to two types of actors: subjects that use contact tracing apps and, except for cases in which only self-reported data are used [34,36], medical professionals that digitally attest to positive cases [23,25,32]. Through their mobile devices, subjects obtain environmental signals from passive sensors [33], proximity signals from active sensors [23,31,32,34,36-38], or a combination of both [25,27,35,36]. The data received are converted into a target format to be stored on the blockchain, the particulars of which are a key differentiator between protocols. While some protocols disregard information privacy [33], more commonly, the confidential nature of tracing data is recognized and addressed by proposing formats that are deemed to prevent a subject’s privacy from being compromised.

The goals of privacy-focused blockchain architectures are as follows: preventing manipulation of diagnostic data [23], preventing impersonation of health authorities [31], protecting the identity of infected persons [25,36], or, most commonly, precluding mass surveillance through the derivation of movement or contact profiles from stored data [25,31,32,34,35,37,38]. To approach these goals, various models to capture data relevant to contact tracing are proposed. Contact data commonly come in the form of contact lists using one-time pseudonyms [23], pseudonymized user data combined with encrypted location information [25], pseudonymized or encrypted diagnoses [31,32], or encrypted epidemiological data [37].

Irrespective of the format, after being generated on the user’s device, contact data are sent to the blockchain. This can happen via submission of a transaction to a public network [23,25,31,33-36,38] or, specifically in the case of private systems where subjects do not have direct access to the blockchain [27,32,37], by passing through an upstream application programming interface that can be operated by government bodies [27,37] or by a consortium of otherwise trustworthy entities [32]. The data are then replicated between nodes. In the case of a positive diagnosis for a subject, there are two alternative patterns. First, subjects can reveal their positive status on the chain proactively, oftentimes by providing some form of proof [23,32]. Second, a pseudonymized diagnosis can be uploaded to the chain [31,33] or endorsed on-chain by an authorized diagnostician [25]. Subjects can then query the blockchain at intervals to obtain a risk assessment based on their previous contacts [32,38] or to receive notifications [23,36,37]. The overall purpose of these proposed systems is to enable decentralized networks that can share trusted data relevant to contact tracing efforts, including self-reported data and environmental signals. Nevertheless, parameters around data storage, computation, and measures to ensure privacy-preserving processing vary and can be further modulated by developers should these be implemented.

### Evaluation of Applicability of Blockchain to Algorithmic Contact Tracing

#### Overview

Based on our review of proposed blockchain system designs for algorithmic contact tracing, we now conduct an in-depth assessment of the potential suitability and technological feasibility for their application to COVID-19 based on a technical evaluation framework. We used the Lo et al [69] framework, which assesses the suitability of applying blockchain for the requirements of general use cases by posing a set of seven questions and associated decision gates to answer the question of whether blockchain or conventional databases are more suitable for a particular technology use case. These questions include the following:

1. Are multiple parties required?
2. Is a trusted authority required?
3. Are operations centralized?
4. Is data transparency or confidentiality required?
5. Is the integrity of transaction history required?
6. Is data immutability required?
7. Is high performance required?

Below, we assess core blockchain features, such as decentralization, information privacy, immutability, data integration, transaction verification, and network performance, aligned with the suitability assessment and applied to the use case of algorithmic contact tracing (see Table 1).

**Table 1:** Suitability evaluation of the applicability of blockchain technology to algorithmic contact tracing, with comparison to conventional database applications (CDAs).

Consideration for blockchain use cases	Evaluation	Indicated system architecture
Are multiple parties required?^a^	Yes	Blockchain is preferred, but CDA is also applicable
Is a trusted authority required?^a^	Yes	Either blockchain or CDA
Is the operation centralized?^a^	Inconclusive	Possibly CDA, as it inherently supports centralized operations
Is transparency required?^a^	No	CDA
Is transaction history required?^a^	No	CDA
Is immutability required?^a^	No	CDA
Is high performance required?^a^	Inconclusive	Possibly CDA, as it can generally achieve higher throughput
Is integration with other systems required?	Yes	Either blockchain or CDA
Is decentralized data validation required?	No	CDA
Is high data reliability required?	Yes	CDA, as it can generally provide higher reliability of data without need for on-chain and off-chain approaches needed for blockchain

^a^This consideration is based on the framework proposed by Lo et al [69].

#### Multi-Party Decentralization

The first question Lo et al [69] raise in their framework is whether a use case requires multiple parties to be involved and, if so, whether a trusted authority is required and whether that trusted authority is decentralizable. In this context, assessing whether there is a need to operate a multi-party decentralized authority on a public or private blockchain is the first topic that needs to be addressed. Public blockchains were conceived as a design paradigm that is effective in an adversarial environment in which no central trusted party exists, and where potential malicious writers operate on the same hierarchical level as honest ones [11]. In this sense, public blockchains constitute fully decentralized networks that do not require a single trusted authority to validate transactions. Private blockchains introduce some variation to this paradigm by limiting who can access a network and, in the case of private permissioned blockchains, by limiting who can participate in the consensus protocol on the network. Hence, private blockchains, and iterations of consortium blockchains—where a single entity or group controls access to the blockchain—inherently exhibit a lower degree of decentralization. Still, either paradigm can only exploit its respective strengths where there is distrust between those who write the data and where trusted third parties are absent [70].

The environment in which algorithmic contact tracing is conducted, however, is very different. Even though it is a multi-party environment, it requires a trusted authority (Question 2) to be involved in the validation of critical public health data, particularly in the context of addressing a pandemic. Decentralizing the role of the trusted authority may not bring with it any added benefit. For example, a multi-party public blockchain network, where patients have the same rights and responsibilities—including access to and validation of data—as medical practitioners and health authorities, is not optimal for case detection and investigation, as other potential nodes participating in the blockchain may inherently be less trustworthy. Specifically, contact tracing is generally carried out in an environment with clear hierarchies, expertise, and legal mandates that national authorities lead [56]. Authorities also supervise the reporting of case numbers to other local, national, and international organizations and develop necessary calculations based on the epidemiology of the disease to assess the risk of transmission associated with the date and duration of contact with an infected individual. The hierarchical nature of this public health use case becomes particularly evident when considering the possibility of intervention by law enforcement against individuals who do not comply with public health measures [71]. Health authorities are, therefore, in control of virtually all of the factors contributing to the technical success of an algorithmic contact tracing regimen, fundamentally making it a centralized problem requiring a trusted authority and centralized operation (Question 3), which may make it more suitable for conventional information management systems. Those systems commonly consist of infrastructure built around relational database management systems and application settings [72] that employ access control mechanisms as mandated by legislation and regulations [73].

#### Information Privacy

Algorithmic contact tracing, while having the potential to be an effective tool for controlling disease transmission [74], has also been characterized, fairly or unfairly, as a potent mass surveillance tool, leading to the fear of the normalization of state-run electronic surveillance [32,75-78]. This can be explained by the nature of the data needed for algorithmic contact tracing, which, as discussed earlier, can manifest as location or contact data. Clarke and Wigan [79] discuss why location data are particularly vulnerable by identifying specific dangers that arise from their collection. Among other factors, they discuss psychological harm through embarrassment, the danger of profiling and suspicion generation through the discovery of behavior patterns, as well as social, cultural, scientific, and economic harm arising from the knowledge or suspicion of being watched [79]. While an in-depth debate of these issues is beyond the scope of this viewpoint, we discuss the influence of blockchain on information privacy by comparison to conventional, centrally managed contact tracing systems. This topic aligns with the blockchain suitability framework’s question that is focused on the tension between weighing the benefits of enhanced transparency against the needs of such systems to maintain confidentiality (Question 4) and the impact of these decisions on data governance and network performance.

When operated on a public network, blockchain poses significant challenges for engaging with privacy-sensitive data, including protected health information. While proposed blockchain-based systems for contact tracing commonly address the privacy of tracing data through cryptographic protocols [25,31,32,34,35,37,38], their effectiveness in an adversarial environment has to be approached with concern for three reasons:

1. Cryptanalysis can bring to light deficiencies in cryptographic protocols previously believed to be secure, potentially revealing data that were believed to be protected from attackers [80].
2. Even protocols that apply data hygiene diligently by “minimizing or eliminating personally identifiable data of...subjects” [81] and appear unproblematic with regard to privacy might be vulnerable to abuse by methods not yet known, potentially through correlation with data from other sources not yet considered [82].
3. The cryptographic integrity of today’s blockchain protocols is threatened by methods of quantum computing [83].

It is, therefore, inadvisable to make any data related to contact tracing, even if considered harmless or undecryptable by today’s methods, available beyond completely trustworthy parties that have a legitimate *need to know*, irrespective of whether data are stored in a conventional or decentralized system.

When operated as a private network, blockchain systems generally have a weak negative effect on information privacy: while more finely grained controls of data access in blockchain are possible through permission structures [84], typically, all nodes in a private blockchain network have visibility of network-wide data. Storing only a hash or a similarly obfuscated datum on-chain and keeping sensitive health-related or individually identifiable data off-chain, including approaches that use off-chain blockchain storage and computation, can improve confidentiality. However, this requires the application of appropriate hash algorithms and randomization techniques [85]. Moreover, obfuscation can diminish the utility of said data and can inhibit network performance, including when data are encrypted [9,69]. Though privacy-preserving approaches to managing health care data leveraging different combinations of off-chain and on-chain storage are possible, their application requires careful design and mapping to appropriate legal and privacy frameworks specific to particular health care use cases and types of data [9]. Given the highly sensitive nature of contact tracing data, confidentiality considerations appear to outweigh the benefits of blockchain-mediated distributed trust and transparency.

#### Data Integrity and Immutability

An original principle and key value proposition of blockchain systems is their ability to provide data integrity and immutability through creating provenance by linking of transaction blocks [11], which means that data appended to the blockchain cannot be deleted or changed trivially and can, therefore, be considered final in most circumstances [86]. While alternative designs providing mutability have been proposed [87], the applications discussed here consider blockchain as a near-immutable technology and emphasize this quality. This aligns with key decision points in the assessment of suitability for use cases (Questions 5 and 6). Immutability has practical disadvantages in an algorithmic contact tracing context since data cannot be expunged after the incubation period. This means that contact records that no longer serve the purpose of enabling contact tracing may still be present in such systems, potentially threatening the privacy of those that reported them or negatively impacting blockchain system performance (Question 7).

Further, proposed applications commonly embrace the *tamper-proof* nature of contact tracing data on the blockchain. This is largely due to the abstract threat of an attacker tampering with tracing data or the risk of having a trusted authority as a single point of failure. However, in the context of digital contact tracing, data integrity and immutability are of less concern than accuracy and correctness, which are decisive factors for predicting chains of transmission. By their nature, data on confirmed cases should come from trusted centralized sources (eg, health authorities). Therefore, the need to establish data provenance by ensuring the integrity of the transaction history through establishing consensus system-wide is rendered of low importance. Unlike other health care use cases, such as enabling enhanced track and trace of pharmaceuticals in the global supply chain, contact tracing data are not a physical asset that requires tracking changes to its access, ownership, and transfer [88].

As discussed, common blockchain protocols aim to achieve immutability of data recorded [89] and, should the need for correcting existing records arise, address it by appending updated records to the blockchain. This stands in contrast to centralized data storage systems in which records can simply be deleted or corrected. Consequently, data hygiene is hard to achieve in blockchain-based algorithmic contact tracing systems, as those might retain tracing data for longer than medically necessary, simply because the technical capabilities to delete them are not given. Incorrect or inconsistent testing results, or duplicates occurring during integration and consolidation of contact tracing data from different jurisdictions and agencies, are equally harder to correct in an immutable setting [69].

#### Performance

Performance (Question 7) is recognized as one of the major challenges for real-world implementation of blockchain systems [90]. This can be attributed to challenges associated with their scalability [91], particularly in the context of modulating between on-chain and off-chain storage and computation [9]. Although scale has been achieved in some blockchain applications in the financial sector by applying partitioning [92] or second-level protocols like *side chains* [93], performance may be negatively impacted by the need to achieve consensus among network members during record creation. Achieving consensus is a complex problem to which different blockchain protocols offer different solutions with varying performance characteristics [94]. In the context of algorithmic contact tracing, throughput (ie, the number of transactions that can be executed per unit of time) can be considered the most relevant approximation of overall performance. What all consensus protocols have in common is that coordination among nodes or members is required, which imposes penalties on throughput in exchange for distributed networks of shared trust. Penalties are particularly severe on major public permissionless blockchains, where data validation and replication are subject to *proof-of-work* or *chain-based proof-of-stake* consensus protocols that are characterized by allowing a throughput of only tens of transactions per second [95].

For example, while permissioned blockchains can provide significant performance benefits over their permissionless counterparts [96], achieving around 1000 transactions per second in some common configurations [97], they are still inferior to traditional replicated databases, particularly when multi-leader strategies with low consistency levels are applied, as these can support throughputs above 15,000 operations per second even under challenging workloads [98]. Therefore, traditional database systems can more effectively address the use case of contact tracing in which data validation, where necessary, can be performed centrally by the appropriate authority. Throughput is critical in the context of algorithmic contact tracing infrastructures, especially where vast populations generate large volumes of contact data rapidly and where privacy requirements will inevitably require off-chain storage and computation. Despite the lack of a standardized workload that would be necessary to conclusively answer this question, it can be speculated that private blockchains may have the capability of handling contact tracing data volumes, at least on a regional scale. Nevertheless, the fact that they do not provide a throughput benefit over traditional database systems minimizes their suitability from a performance perspective.

#### Other Evaluation Considerations

In addition to the evaluation based on the framework proposed by Lo et al [69], further aspects are relevant for assessing blockchain’s suitability for enhancing algorithmic contact tracing. These aspects include data integration, transaction verification, and data reliability as discussed in this section.

The further processing of data gathered via algorithmic contact tracing is largely a problem of data integration (ie, one of combining tracing data with data from different sources, for example, diagnostic data from COVID-19 testing centers and clinical data from EHRs). Here, blockchain can provide benefits by defining a standardized format for transaction data payloads and standard processing logic via smart contracts. Efforts to address data integration are underway, as exemplified by emerging standards at the intersection of blockchain and pandemic and epidemic surveillance [99]. There are, however, challenges concerning the integration with existing health record management systems, such as the cost of change incurred [100], ensuring regulatory compliance of an integrated information technology environment [101], dealing with privacy and confidentiality policies specific to health information (eg, implications of the Health Insurance Portability and Accountability Act in the United States [102] and the General Data Protection Regulation in the European Union [103]), or the immaturity of proposed standards that is detrimental to achieving interoperability [104].

*Smart contracts* [14,105] constitute agreements that are executed without the involvement of the concerned parties as part of a blockchain protocol. They are a key component of many distributed applications and can be implemented in various programming languages following different paradigms that come with various security features [106]. The common element is that they allow application developers to encode the logic that governs what constitutes a legitimate transaction on the blockchain. Such logic can validate endorsement policies and rules concerning data integrity, thereby ensuring the added content’s correctness. Rules around data audit and system access that in centralized systems are commonly enforced by role-based access control mechanisms [107] could also be executed through smart contracts [108]. Conceivably, in the case of a contact tracing app, smart contracts could be developed by a trusted vendor and audited by a credible authority. Further confidence in its correct execution could be gained through formal verification [109]. Still, even in a flawless implementation, smart contracts can only be exploited partially here. While they provide value as a means of ensuring that confirmed case data originate from trusted sources and have not been tampered with, most contact tracing data are user generated and based on signals from outside of the system (eg, pseudonyms of devices in close proximity or geographic locations). For such data, a smart contract differentiating between a legitimate data set and an illegitimate one can, at best, be heuristic. This diminishes the usefulness of the transaction verification capabilities provided by blockchain technology.

Data reliability can be considered essential in contact tracing, where a loss of recent contact data could lead to participants at risk of infection going unnotified. Nodes on a public blockchain network can leave and join at will without risking data loss. Commonly, data are fully replicated between existing nodes and those who join the network. This technique allows for high degrees of redundancy, especially on public blockchains where the number of replicating nodes can be very large (eg, up to 23,000 in the case of Bitcoin [110]). Private blockchain deployments can run in arbitrary topologies, which makes the degree of redundancy they provide contingent on the configuration chosen by the designer of the system. Redundancy positively influences availability, as clients can select an alternative replica to interact with, in case of failure. This is, however, not a unique benefit of blockchain. High reliability can also be achieved via more traditional centralized data replication protocols used in the context of cloud computing, where data redundancy levels are often configurable [111].

## Conclusions

Blockchain, although not in productive use in this context, has increasingly been discussed as a technology to support algorithmic contact tracing efforts targeting COVID-19. A question resulting from this trend is whether this technology can replace or enhance the centralized architectures that are operational today. To address this question, we examined blockchain-based contact tracing concepts discussed in the literature. Upon realizing similarities in their design, we derived an archetypal system architecture capturing their common characteristics. Subsequently, guided by an evaluation framework, we explored the potential benefits of this system architecture over conventional approaches to data storage. The results of this suitability evaluation indicate that blockchain-based protocols as currently presented do not provide benefits significant enough to warrant the prioritization of their implementation. This is primarily due to the incongruity between the centralization of organizational and administrative processes surrounding contact tracing and the decentralized nature of blockchain technology. Further technical arguments in support of this result are concerns about the impact of blockchain on the privacy of personal data, unclear benefits of blockchain’s key features (ie, enhancing transparency, data provenance, and immutability), the challenges around integrating blockchain systems with existing sources of the health dataverse in legally compliant ways, and a lack of performance benefits over conventional information management systems. The result of the suitability analysis is reinforced by the fact that conventional, centralized, algorithmic contact tracing systems are already integral parts of the pandemic mitigation strategies of some of the countries that are most successful in controlling the spread of COVID-19. Instead of focusing on algorithmic contact tracing, future efforts to leverage blockchain technology in the fight against COVID-19 could turn to the assessment of other promising use cases for suitability. Health supply chain management, digital immunization passports, and the management of digital identity in the context of COVID-19 patient journeys are areas where blockchain might be more appropriate, not least because investments in technology infrastructure and stakeholder buy-in are more mature here.

## Abbreviations

ASCLEPIOS: Advanced Secure Cloud Encrypted Platform for Internationally Orchestrated Solutions in Healthcare
EHR: electronic health record
